# Manufacturing Natural Killer Cells as Medicinal Products

**DOI:** 10.3389/fimmu.2016.00504

**Published:** 2016-11-15

**Authors:** Christian Chabannon, Bechara Mfarrej, Sophie Guia, Sophie Ugolini, Raynier Devillier, Didier Blaise, Eric Vivier, Boris Calmels

**Affiliations:** ^1^CBT-1409: INSERM, Aix Marseille Univ, Institut Paoli-Calmettes, AP-HM, Marseille, France; ^2^CRCM: INSERM, CNRS, Aix Marseille Univ, Institut Paoli-Calmettes, CRCM, Marseille, France; ^3^UM2, INSERM, Centre d’Immunologie de Marseille-Luminy, U1104, CNRS UMR7280, Aix-Marseille University, Marseille, France; ^4^Laboratoire d’Immunologie, Hôpital de la Conception, Assistance Publique – Hôpitaux de Marseille, Aix-Marseille University, Marseille, France

**Keywords:** natural killer cells, innate lymphoid cells, immunotherapy, immuno-oncology, cellular therapy, cell transplant, manufacturing

## Abstract

Natural Killer (NK) cells are innate lymphoid cells (ILC) with cytotoxic and regulatory properties. Their functions are tightly regulated by an array of inhibitory and activating receptors, and their mechanisms of activation strongly differ from antigen recognition in the context of human leukocyte antigen presentation as needed for T-cell activation. NK cells thus offer unique opportunities for new and improved therapeutic manipulation, either *in vivo* or *in vitro*, in a variety of human diseases, including cancers. NK cell activity can possibly be modulated *in vivo* through direct or indirect actions exerted by small molecules or monoclonal antibodies. NK cells can also be adoptively transferred following more or less substantial modifications through cell and gene manufacturing, in order to empower them with new or improved functions and ensure their controlled persistence and activity in the recipient. In the present review, we will focus on the technological and regulatory challenges of NK cell manufacturing and discuss conditions in which these innovative cellular therapies can be brought to the clinic.

## Introduction

Cellular therapies are, nowadays, increasing in numbers and in diversity. Manufacturing of various types of immune cells is likely to provide additional therapeutic resources and complete the portfolio of immunotherapies, along with chemical molecules and engineered monoclonal antibodies. It is envisioned that combination of these different medicinal products, tailored to disease characteristics as well as to the host (immune) environment (1), will contribute to precision medicine and expectedly to higher rates of success in the cure of a variety of health disorders, including, but not restricted to, cancers. Among immune effectors amenable to cell and genetic manipulation prior to adoptive transfer, natural killer (NK) cells present with appealing biological characteristics. NK cells are innate lymphoid cells (ILC) and contribute to innate immunity (2). Their activities are regulated through the biological modulation of a large array of both inhibitory and activating receptors, including killer cell immunoglobulin-like receptors (KIR), NKp44, and NKp46. These receptors do not bind specific antigens on target cells as do T cells, but rather molecules induced by cellular stress that provide an activating signal, or human leukocyte antigen (HLA) molecules that predominantly provide inhibitory signals; already published material provides in-depth description of these pathways. Here, we review the existing literature that describes the rationale for various technological approaches to NK cell manufacturing, either autologous or allogeneic, as a prerequisite to adoptive transfer and clinical evaluation of these peculiar populations of immune effectors.

## Medical Applications of NK Cell Manufacturing and Adoptive Transfer

### Autologous NK Cells

**Figure 1:**
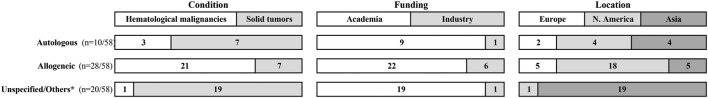
**Ongoing clinical trials that evaluate NK cell-based cellular therapies**. Data were collected at https://clinicaltrials.gov/ in September 2016, using the search terms: “recruiting studies,” “NK,” or “natural killer” in the “Title” field. Raw data are available in Data Sheet S1 in Supplementary Material. *Unspecified = 18 studies with no designation whether NK cells are autologous or allogeneic; others = 1 study tests NK cell line and 1 study compares autologous to allogeneic NK cells.

Adoptive transfer of NK cells engineered to express new or augmented functions represent an interesting avenue for the treatment of various high-risk malignancies in which conventional options have failed (Figure 1). Examples include B-chronic lymphocytic leukemia (B-CLL) (3), multiple myeloma (4), and also tumors of non-hematopoietic origin such as breast cancer (5), melanoma, or renal cell carcinoma (6).

Early studies reporting the adoptive transfer of CD56^+^ bead-selected autologous NK cells into patients with metastatic cancers, in combination with high doses of IL-2 demonstrated feasibility yet poor clinical efficacy (7). Patients experienced severe toxic side effects due to the high doses of IL-2. In addition to activation-induced NK cell death, NK cell function may have been inhibited due to regulatory T cell expansion in response to high IL-2 doses (8, 9). Reducing the daily IL-2 doses after NK cell transfer resulted in limited clinical success (10). Several strategies are under investigation to overcome this hurdle [reviewed in Ref. (11)]. Another mechanism by which autologous NK cells are inhibited is by self-HLA molecules. Therefore “releasing the brakes” with anti-KIR antibodies such as Lirilumab^®^ that targets the inhibitory KIR receptors on NK cells could be one approach (12–14).

### Allogeneic NK Cells

A number of arguments support an important role for donor-derived NK cells in the context of allogeneic hematopoietic stem cell transplantation (allo-HSCT). Following the administration of either myelo-ablative (15) or reduced-intensity (15–17) conditioning regimen and allo–HSCT, the rapid reconstitution of high numbers of circulating and phenotypically defined NK cells is associated with better clinical outcome. The recovery of various functions for donor-derived cells may be further modulated and improved *in vivo* with additional intervention (18).

Transplantation of high doses of immune-selected CD34^+^ cells collected from haploidentical donors after myelo-ablative conditioning regimen has provided a setting which demonstrates that “KIR-incompatibility” was associated with lower incidence of disease relapses, at least for AML (19). Transplantation of T-replete marrow or blood cell grafts obtained from haploidentical donors, using modified immune-suppressive conditioning regimen such as those including posttransplant cyclophosphamide, represent a more widely applicable procedure, in which to further explore the potential contribution of alloreactive NK cells in posttransplant clinical events. Unexpectedly, a recently published report suggests that, in this context, the presence of recipient class I ligands to donor KIR receptors confers some protection to the recipient against leukemia relapse, an observation that needs further confirmation and would imply a role for killer activating receptors (KAR) as much as for KIR (20). The role of alloreactive NK cells remains more elusive in the context of HSCT performed from other categories of donors. Expression of specific KIR receptors in HLA-matched unrelated donors was demonstrated to produce superior or inferior clinical outcomes in recipients, depending on donor–recipient combinations (21–23).

Adoptive transfer of allogeneic NK cells either with a stem cell graft *ex vivo* depleted of immune effectors or as a substitute to posttransplant “donor lymphocyte infusions” (DLIs) is thus appealing as a way to improve engraftment, immune reconstitution, and antitumor activity with reduced chances of triggering graft-versus-host disease (GVHD) (24). Results of a small number of clinical trials have been reported so far, demonstrating the feasibility of manufacturing allogeneic NK cells from matched related, matched unrelated, or mostly from haploidentical donors (25–29). Although allogeneic NK cell infusions were generally reported as safe, a recent publication describes the clinical outcome of a small cohort of pediatric patients treated for non-hematological high-risk malignancies and a high proportion of aGVHD triggered by HLA-matched donor-derived NK cells (30). Mostly, these limited clinical results suggest that additional improvements are needed either during the *ex-vivo* manufacturing process (31) or after infusion of manufactured NK cells (25) to improve long-term persistence and activity *in vivo*.

## Factors Affecting NK Cell Product Manufacturing

Many variables contribute to an efficient NK cell generation protocol (Table 1). Donor–recipient combinations, the source of starting material and culture conditions are factors that must be carefully selected to optimize the manufacturing process and potentially the clinical efficacy of the resulting medicinal product upon administration to the recipient.

**Table 1:** **Factors affecting the ouctome of the manufacturing process of NK cell-based medicinal products**.

**Cell source**
Bone marrow
Umbilical cord blood
Embryonic stem cells
Induced pluripotent stem cells
NK cell lines
**Culture conditions**
Cytokines (IL-2, IL-15, IL-12, IL-18)
Feeder cells (autologous PBMC, EBV-TCT-LCL, K562-mb15-41BBL)
Antibodies (anti-CD3, anti-CD52)
Genetic manipulation (retro- or lentiviral-based transduction, mRNA transfection)
**Culture containers**
Standard culture flasks
Culture bags
Gas-permeable static cell culture flasks
Bioreactors
**Final product evaluation**
Viability (live/dead)
Identity and contamination (CD56, CD16, CD3, CD14, CD45, CD19)
Yield
Phenotype (KIR, NKp44, NKp46, NKG2A, NKG2C)
Functionality (degranulation, cytokine release, target cell lysis, activation)

### Donor Selection

In the setting of allogeneic NK-DLI, donor selection can affect the clinical outcome of NK cell therapy, since certain KIR, HLA, and FcγR polymorphisms influence NK cell function (32, 33). KIR typing can be genotypic, classifying donors on the basis of gene expression of activating and inhibitory KIR (34), thereby assigning them scores to select “preferable donors” (35–37). Additionally, KIR typing can be phenotypic, assessing surface protein expression of KIRs (38), adding another layer of complexity to the selection of “preferable donors.” KIR “allele-typing” is a recent addition to the donor selection algorithm, whereby alleles that possess better functional properties (stronger licensing capability and more durable surface expression upon ligand binding) are favored (22, 39). Typing FcγR polymorphism is relevant in NK cell therapy settings that use monoclonal antibodies to enhance NK cell activation and consequently empower their antibody-dependent cytotoxicity (ADCC) properties (40, 41). All these strategies have helped define “preferable donor” profiles.

### Source of Cells

Natural killer cell therapies can be manufactured from a variety of sources: these include peripheral blood, either steady-state or taking advantage of apheresis performed to collect hematopoietic stem and progenitor cells mobilized with growth factors such as granulocyte colony-stimulating factor (G-CSF), bone marrow, and cord blood.

#### Peripheral Blood Mononuclear Cells

Peripheral blood mononuclear cells (PBMC) can be collected in large numbers using apheresis. Nowadays, it is the preferred source for allo-HSCT; donor apheresis is collected after receiving a mobilization treatment that increases the percentage and number of circulating progenitor and stem cells (as evaluated by the number of circulating CD34^+^ cells); G-CSF is the only marketed agent for CD34^+^ cell mobilization that can be used in donors; the use of other mobilizing agents such as acutely myelo-suppressive drugs or plerixafor is restricted to patients undergoing autologous collection. Since CD34^+^ cells represent only a small proportion of collected PBMC, these collected cell products may also represent a source of immune effectors, and thus either an alternative to PBMC collected in homeostatic conditions for standard DLI or a starting material for further immune cell manufacturing, including NK cell manufacturing. One caveat to this approach is that studies looking at the effects of G-CSF on NK cell function have produced controversial results; some studies suggest minimal consequences (42, 43), while others suggest significant changes (44).

Non-mobilized apheresis products contain 5–15% NK cells. To isolate NK cells, the strategy commonly used is CD3^+^ cell depletion of PBMC, followed by CD56^+^ cell enrichment using immune-magnetic bead separation with medical devices and clinical-grade reagents.

#### Bone Marrow

Since bone marrow is nowadays used as the source of stem cells for a minority of recipients – in part due to the increased resource needed for the logistics of BM collection compared to aphereses – there is little preclinical or clinical experience in the manufacturing of NK cells from this starting material.

#### Cord Blood Cells

The use of cord blood (CB) as a source of stem cells has raised great hope in the field 30 years ago, when the first clinical transplants were reported (45). Cord blood unit (CBU) can be used even when not fully matched to the recipient, offering the opportunity to identify a “donor” even for patients who had no HLA-matched related or unrelated donor. CB transplantation is nowadays facing tough competition from the rapidly emerging field of related haploidentical transplantation, and is further hampered by the lengthy immune reconstitution and the lack of possibility to use pre-emptive or curative DLI posttransplant.

However, more than 600,000 CBU that are stored in public banks worldwide – not to mention the unknown cumulative number of CBU preserved in private CB banks – represent a unique source of human material to start with the manufacturing process. Indeed, preclinical validation studies reported the production of significant numbers of functional NK cells either from a complete CBU (46) or even from a minute sample of a CBU (47), the latter opening the way to posttransplant immune cellular therapies in recipients of CBU transplantation while the former offers the promise of “off-the-shelf” allogeneic NK cellular therapies.

Initial attempts to use CD56^+^ bead-selected NK cells from CB followed by culture on mesenchymal stromal cells in the presence of cytokines resulted in modest yields incompatible with therapeutic needs (48). Starting from immune-selected CB CD34^+^ cells and using refined protocols produced more interesting results (49–51). Such efforts culminated in a novel good manufacturing practice (GMP)-compliant technique that mimics the extracellular bone marrow environment: stromal cell-free/serum-free medium, heparin, and cytokine supplements (52, 53). Consequently, Glycostem Therapeutics (Oss, the Netherlands) and Radboud University Medical Center (Nijmegen, the Netherlands) are currently conducting a Phase I/II clinical trial in elderly AML patients using NK cell products generated using this method (CCMO nr. NL31699 and Dutch Trial Register nr. 2818) (see Figure 1 and Data Sheet S1 in Supplementary Material for a list of ongoing clinical trials).

#### ES and iPS Cells

Manufacturing of clinical-grade NK cells from either embryonic (ES) or induced pluripotent stem (iPS) cells nowadays appear as a futuristic option, although preclinical demonstrations that NK cells can be differentiated from these sources of pluripotent stem cells were already published (54–56). For iPS cells, several factors affect the pluripotency and differentiation abilities of reprogramed cells: the choice of target donor somatic cell type and the reprograming protocol, including the nature and combination of genes as well as the method used to deliver transcription factors into somatic cells (57).

An important step in the specific hematopoietic lineage-differentiating protocols starting from ES or iPS cells is the generation of CD34^+^ hematopoietic precursors, particularly CD34^+^CD45^+^ cells, preferred for their high content in hematopoietic progenitors (58). A 30-day culture protocol of sorted ES cells-derived CD34^+^ cells together with feeder cells (murine fetal-liver-derived stromal cell line) and cytokines generates NK cells with typical maturation markers and target cell lysis capabilities (58, 59). Another expansion method has been described for ES or iPS cells, using an embryonic body assay followed by culture with feeder cells and cytokines (60, 61).

Similar to what has been mentioned for CBU, progress in the development of safe, efficient, and standardized clinical-grade manufacturing protocols will offer an opportunity to develop off-the-shelf personalized and non-immunogenic cellular therapies.

#### NK Cell Lines

*Ex vivo*-expanded primary NK cells persist *in vivo* for short periods of time after adoptive transfer. In an attempt to take advantage of the long lifetime of established cell lines, several groups have evaluated their therapeutic potential. Although other cell lines exist (NKG, YT, NK-YS, YTS cells, HANK-1, and NKL cells), the NK-92 cell line (NantKWest Inc., Culver City, CA, USA) characterized by good cytotoxicity and expansion kinetics (62, 63) has been predominantly evaluated in preclinical investigations and clinical trials (NCT00900809 and NCT00990717) (64). It has been tested in a small number of clinical contexts, yet with minimal efficacy (65–67). Recently, chimeric antigen receptor (CAR) modification by gene transfer for NK cells has opened a new avenue to explore (68, 69). NK cell lines represent a more homogeneous population for CAR modification, compared to peripheral blood NK cells; however, this advantage is largely offset by the need to additionally transfect CD16 to gain ADCC function and the necessary irradiation before infusion for safety reasons, rendering them unable to expand *in vivo*. Choice of the CAR construct adds another layer of complexity (69).

### Culture Conditions: Medium, Cytokines, and Cell Culture Systems

As already described, NK cells are generally isolated through immune-selection techniques, using the canonical CD3^−^/CD56^+^ phenotype (42, 70), then cultured for functional activation and possibly expansion. Furthermore, NK cells can be genetically engineered to express natural or chimeric molecules empowering them with improved immune functions (5, 64).

Expansion and activation of potent cytotoxic NK cells require several signals for survival, proliferation, and activation. Culture conditions, thus, incorporate media and serum supplements, together with clinical-grade cytokines, monoclonal antibodies, or other soluble molecules, and possibly native or engineered cell feeders. Culture conditions can further be improved through the substitution of bioreactors to static conditions (Table 1).

As already mentioned, and since most protocols that use only cytokines result in limited NK cell expansion, the introduction of feeder cells in the culture protocol has been extensively tested. Feeder cells provide additional stimulatory signals necessary for NK cell proliferation. Monocytes provide humoral signals and cell-to-cell contacts hence can serve as feeder cells (71); irradiated autologous PBMC have been used as feeder cells to produce sufficient numbers of NK cells with acceptable purity (6, 72, 73). Alternatively, irradiated allogeneic cells have been evaluated: Epstein–Barr virus-transformed lymphoblastoid B cell lines (EBV-TM-LCL), K562 cells (leukemic cell line) engineered to express a membrane-bound form of IL-15 fused to the T-cell receptor CD8α and the 41BB ligand (74–78), or K562 cells transduced with IL-21 (79). Such feeder cells proved effective in preclinical validation of the production of clinically relevant numbers of NK cells, however, raise regulatory issues when it comes to manufacture medicinal products.

Since the presence of residual feeder cells in the final product is of major concern for clinical applications (80), alternative approaches are evaluated as substitutes. Anti-CD3 (OKT3) antibodies in addition to IL-2, with or without IL-15, produced substantial although lower fold expansion of CD3^−^/CD56^+^-enriched cells (81–86) than protocols that use feeder cells.

Expansion strategies of clinical-grade NK cells usually require 7–21 days of culture; up to 28 days of culture have been reported (87). There is an incentive to substitute animal or human serum-replete medium with animal and human component-free medium. The most commonly used media for CB-derived NK cells is glycostem basal growth medium (Clear Cell Technologies, Beernem, Belgium), preferred for being free of animal-derived components (52, 53). For PBMC-derived NK cells, preferred media are: X-Vivo™ serum-free media (Biowhittaker, Verviers, Belgium), AIM V^®^ serum-free medium (Thermo Fisher Scientific, Grand Island, NY, USA), Stem Cell Growth medium (Cell Genix, Freiburg, Germany), or complete Roswell Park Memorial Institute 1640 (Biowhittaker, Verviers, Belgium).

Media supplements still being used by some groups include GMP-grade human AB serum, pooled human AB plasma, or fetal bovine serum (FBS). GMP-grade cytokines (recombinant human IL-2 and IL-15), antibodies (anti-CD3-OKT3), and other ancillary reagents (nicotinamide-NAM) commonly serve as medium supplements for PBMC-derived NK cell generation. Additional growth factors and cytokines are necessary for CB-derived NK cells since the starting material is commonly CD34^+^ stem cells [stem cell factor (SCF), IL-7, IL-15, IL-2, IL-6, flt3 ligand (Flt3-L), thrombopoietin (TPO), G-CSF, low molecular weight heparin (LMWH), granulocyte-macrophage colony-stimulating factor (GM-CSF), leukemia inhibitory factor (LIF), MIP1α].

In addition to using T75 cell culture flasks, several groups have used culture bags (Baxter LifeCell^®^ or VueLife^®^) (76, 83). On larger scales, gas-permeable static cell culture (G-Rex^®^) flasks (Wilson Wolf Manufacturing, New Brighton, MN, USA) (78) or WAVE Bioreactor™ (GE Healthcare Life Sciences, Chicago, IL, USA) (83) served as expansion platforms.

## Amplitude of NK Cells Expansion and Definition of an Optimal Cell Dose

Numbers of infused NK cells in clinical trials typically range from 5 to 50 × 10^6^ NK cells/kg, but infusion of as many as 10^8^ NK cells/kg has been reported (88). Based on percentages of NK cells in the starting materials, manufacturing the higher doses implies significant expansion during *in vitro* cultures. This raises a practical issue, since, in the absence of feeder cells, NK cells expansion is modest if any. Using autologous irradiated PBMC as feeder cells, up to 2,500-fold expansion of functionally active NK cells at day 17 has been reported (89). The use of genetically modified cell lines as feeder leads to a 30,000-fold expansion of NK cells after 21 days of culture (79).

A recent study took advantage of the introduction of anti-CD3 and anti-CD52 monoclonal antibodies over a period of 14 days and reports a median 1500-fold increase in NK cell numbers; however, it must be emphasized that T cells represent up to 40% of the final cell product and that NK cells were not obtained through a cGMP protocol (90).

## Quality Controls and Release Criteria for Engineered NK Cell Cells

Tools for assessing the efficacy of NK cell generation protocols are necessary for comparing technical results from different NK cell therapy studies. Furthermore, European Medicine Agency (EMA), Food and Drug Administration (FDA), and several guidelines require the characterization of the final product to define release criteria in order to ensure safety and efficacy.

Basic, yet essential, criteria are generally used to characterize the final product: these include purity and viability of the target cell population, contamination with undesirable cells such as residual T and B cells, and sterility. These are commonly used as release criteria although their relevance may vary for different clinical conditions: T cell contamination for instance is most important in an allogeneic, but not so much in an autologous setting. More sophisticated testing may provide additional information: a reduction in telomere length indicates cell senescence due to extensive long-term culturing.

Phenotype and function (tumor cytotoxicity) are additional characteristics that should help identify the most effective NK cell products. When expanded NK cells were compared with freshly isolated and IL-2-activated NK cells, a higher expression of NKG2D, TNF-related apoptosis-inducing ligand (TRAIL), and natural cytotoxicity receptors NKp30, NKp44, and NKp46 was reported (91), in addition to higher cytotoxicity to K562 cells. Efforts are, however, much needed to harmonize technical protocols and identify a panel of phenotypic and functional biomarkers that would allow comparisons between protocols that evaluate adoptive transfer of NK cells (92). It is essential to mention that such a panel needs to be run within a reasonably short time to release the product in time for a “fresh infusion.”

Cryopreservation and conservation of cytolytic activity of thawed NK cells would render multiple rounds of adoptive NK cell infusions feasible. Lapteva et al. and Berg et al. reported that an overnight activation with IL-2 would rescue the reduced cytolytic activity of thawed NK cells, yet at the cost of a diminished recovery (74, 78). Efforts to optimize cryopreservation and thawing methods are in progress.

## Regulatory Status of Engineered NK Cells and Commercial Perspectives

Autologous and allogeneic NK cells engineered from primary human cells are individually produced for a unique and designated individual, rather than manufactured as batches. Since manufacturing incorporates *ex-vivo* culture and activation of immune-selected cells from the primary material, these will be considered as substantially manipulated or more-than minimally manipulated cell products, and thus will qualify as “advanced therapy medicinal products” (ATMPs) and somatic cell therapy products as defined in EC regulation 1394/2007. Before the regulation was released, such cell therapies were engineered as part of clinical research protocols by cell processing facilities, usually supported and operated by academia, no differently from minimally manipulated cell transplants. Since the regulation has been published, the view is that somatic cell therapy products and gene therapy products will be manufactured in compliance with *good manufacturing practices* (85, 86, 93), and eventually marketed by industry, when a marketing authorization is granted by competent authorities at a European level, i.e., by the EMA or by the FDA in the USA.

Beyond academic investigations, NK cells have now aroused the interest of a significant number of pharma companies (94) (see Figure 1), although no NK-based cellular therapy has so far been authorized as an ATMP in Europe, nor a somewhat comparable status in the USA. However, in December 2014, orphan designation (EU/3/14/1395) was granted by the European Commission to Glycostem, for the GCT-NK cell product, made of allogeneic *ex vivo*-generated natural killer (NK) cells from CD34^+^ CB progenitor cells for the treatment of acute myeloid leukemia (EMA/COMP/730059/2014 Committee for Orphan Medicinal Products).

## Conclusion

Over the last 30 years, enormous progress has been made in our understanding of the biology of NK cells. New agents targeting their activity *in vivo* have been evaluated, and technological improvements in NK cell manufacturing have been introduced in the clinic. However, the demonstration that modulation of NK cell activity by any of these means can achieve therapeutic activity over a wide range of diseases is still awaited. The ability to follow and image *in vivo* adoptively transferred autologous or allogeneic NK cells would represent a major advantage to understand the “pharmacokinetics” and mechanisms of action of these immune effectors, as illustrated in preclinical (95) as well as in clinical (96) studies. It would help to understand the consequences of culture conditions on *in vivo* persistence and activity. Ongoing developments for innovative cellular therapies in the academic sector as well as in the commercial sector suggest that such progress may result in broader clinical applications in the near future.

## Author Contributions

All authors are members of a Marseille-based consortium that aims at translation of scientific discoveries to clinically applicable innovations, with a focus on innovative cellular therapies for patients affected with hematological diseases. All authors have contributed to the writing of this manuscript and had the opportunity to review the draft and final versions.

## Conflict of Interest Statement

The authors declare that the research was conducted in the absence of any commercial or financial relationships that could be construed as a potential conflict of interest.

## Abbreviations

ADCC, antibody-dependent cytotoxicity; ATMP, advanced therapy medicinal product; CAR, chimeric antigen receptor; CB, cord blood, placental blood; CBU, cord blood unit; CLL, chronic lymphocytic leukemia; DLI, donor lymphocyte infusion; EMA, European Medicines Agency; ES, embryonic stem (cells); FDA, Food and Drug Agency; Flt3-L, flt3 ligand; G-CSF, granulocyte colony-stimulating factor; GM-CSF, granulocyte-macrophage colony-stimulating factor; GMP, good manufacturing practices; GvHD, graft-versus-host disease; HLA, human leukocyte antigens; HSCT, hematopoietic stem cell transplantation; IL-x, interleukin x; ILC, innate lymphoid cells; iPS, induced pluripotent stem (cells); KAR, killer activating receptors; KIR, killer cell Immunoglobulin-like receptors or killer inhibitory receptors; LIF, leukemia inhibitory factor; LMWH, low molecular weight heparin; MIP1α, Macrophage Inflammatory Protein-1α; NK, natural killer; PBMC, peripheral blood mononuclear cells; SCF, stem cell factor; TPO, thrombopoietin; TRAIL, TNF-related apoptosis-inducing ligand.
